# Case Report: Primary NK/T Cell Lymphoma Nasal Type of the Colon With Multiple Intestinal Perforations

**DOI:** 10.3389/fonc.2021.577939

**Published:** 2021-03-17

**Authors:** Yan Zhang, Weiping Liu, Xinyue Zhang, Bing Wu

**Affiliations:** ^1^Department of Radiology, West China Hospital, Sichuan University, Chengdu, China; ^2^Department of Pathology, West China Hospital, Sichuan University, Chengdu, China; ^3^Department of Nuclear Medicine, West China Hospital, Sichuan University, Chengdu, China

**Keywords:** Nk/t cell lymphoma, extranodal, perforation, non-Hodgkin lymphoma, Intestinal neoplasm

## Abstract

**Background:** Extranodal NK/T cell lymphoma is a rare non-Hodgkin lymphoma mainly involving the upper aerodigestive tract, even rarer is primary extranasal disease involving the intestine. We present a case of primary intestinal NK/T cell lymphoma with diagnostic challenge, which eventually developed into multiple intestinal perforations.

**Case Presentation:** A 35-year-old man presented with diarrhea and recurrent fever. Abdominal CT revealed multi-segmental intestinal wall thickening. Colonoscopy showed multiple irregular ulcers in colon. During the hospitalization, the patient developed intestinal perforation and an emergency surgery was performed. The resected specimen showed multiple perforations of the colon. The surgical samples underwent pathological analysis, and a diagnosis of extranodal NK/T cell lymphoma nasal type was confirmed. After recovering from surgery, the patient started receiving chemotherapy and PD-1 monoclonal antibody. Fortunately, he was discharged after significant improvement in his general condition. Eleven months follow-up was uneventful.

**Conclusion:** Early diagnosis of primary intestinal NK/T cell lymphoma is frequently difficult. Most patients were definitely diagnosed only after surgical resection following complications, resulting in a poor prognosis. Therefore, doctors should maintain high suspicion of this malignancy for early diagnosis at an early stage clinically.

## Background

Extranodal NK/T cell lymphoma nasal type (ENKTL) is a subtype of non-Hodgkin lymphoma (NHL). It is an invasive lymphoma derived from NK cells or cytotoxic T cells, which is related to Epstein-Barr virus infection. ENKTL is more frequent in Asia, Mexico and Central or South America and more commonly affects males than females (1, 2). ENKTL mainly occurs in the nasal/paranasal area, intestinal involvement is rare. Incidence of primary large bowel lymphomas comprises only 0.2–0.6% of large bowel malignant tumors (3). Diffuse large B-cell lymphoma is the most common histologic subtype of NHL, with ENKTL of the large bowel being less frequent (4). The main complaints, colonoscopy and imaging findings of patients with ENKTL involving the colon are non-specific, and may be similar to many other benign and malignant lesions. Diagnosis depends on immunohistochemistry. In this paper, we describe a very rare case of ENKTL involving the colon with multiple intestinal perforations.

## Case Presentation

A 35-year-old male presented with diarrhea and abdominal pain after eating seafood and drinking 5 months before admission. The abdominal pain could be relieved after defecation. Two weeks later, he began to have a fever, up to 39.8°C, accompanied by chills. He was admitted to the local hospital and broad spectrum antibacterial drugs were started empirically. Despite antibiotics, the fever continued to occur repeatedly, in peaks up to 41°C, and the diarrhea persisted. Then the patient was suggested to be transferred to our institution.

**Figure 1:**
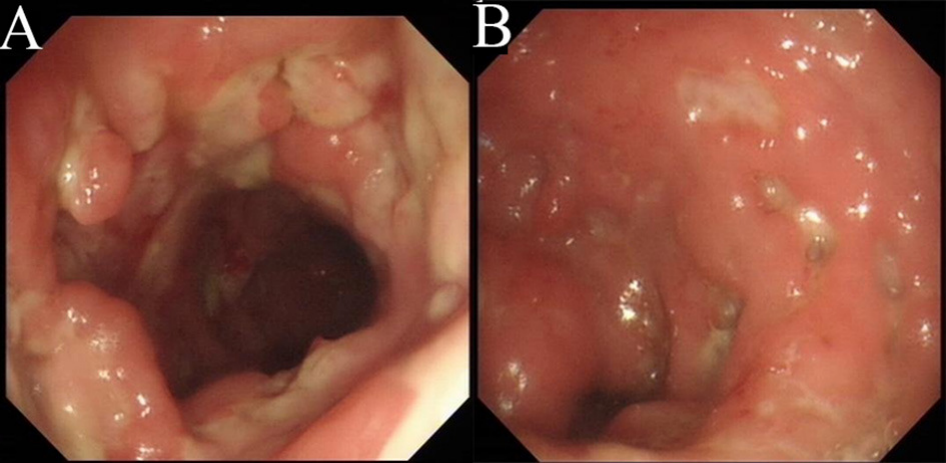
Colonoscopy. There are multiple irregular ulcers in the colon. **(A)** Ascending colon. **(B)** Sigmoid colon.

**Figure 2:**
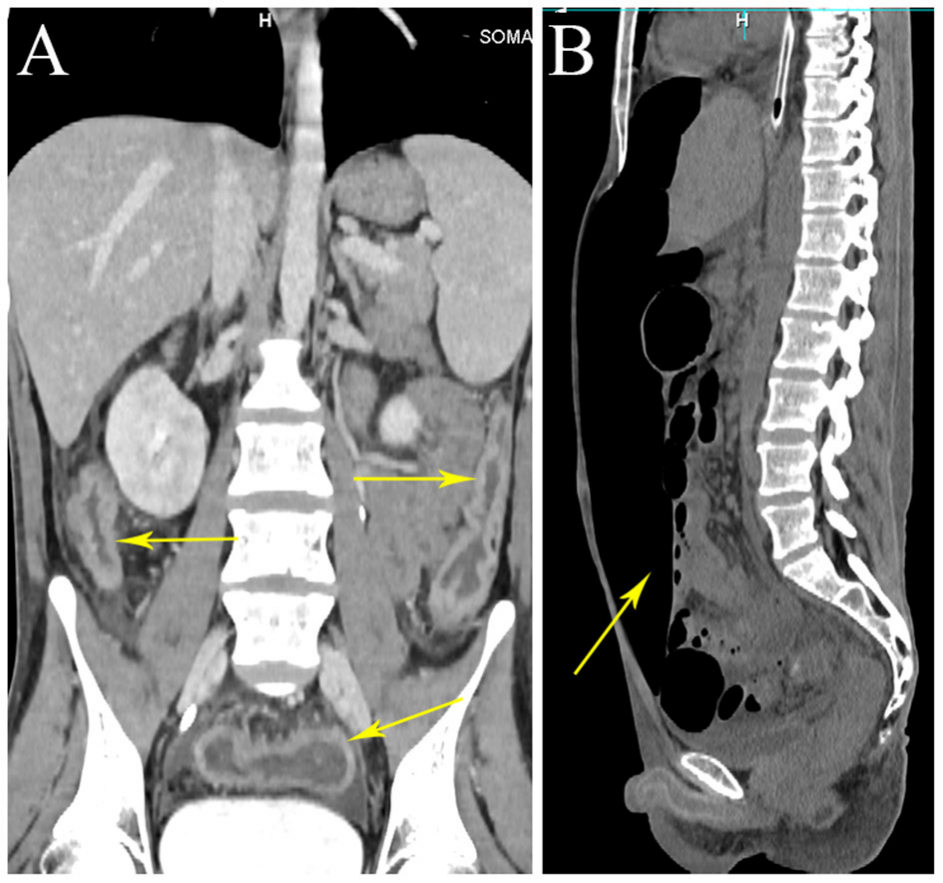
Whole abdominal CT. CT scan showing wall thickening of the colon (arrows). CT scan showing a large amount of free gas in abdominal cavity (arrow). **(A)** Coronal view. **(B)** Sagittal view.

**Figure 3:**
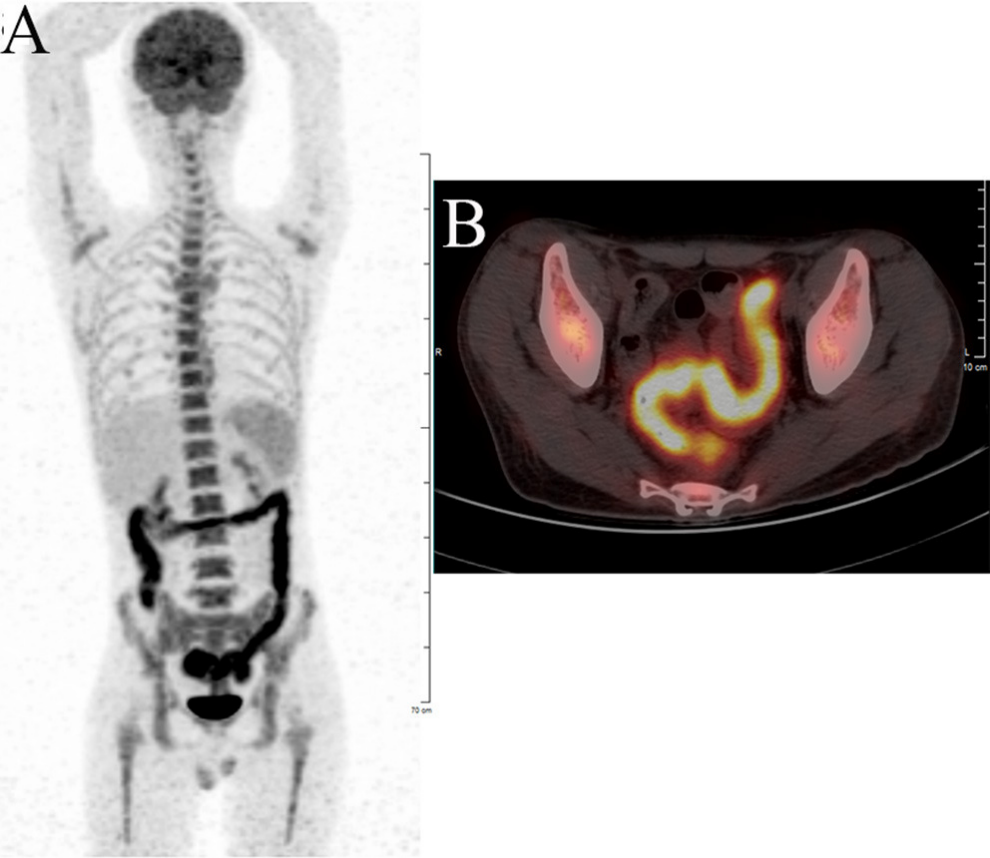
F-18 fluorodeoxyglucose positron emission tomography with computed tomography (^18^F-FDG PET/CT) showing significant increased FDG uptake in the intestinal wall of the whole colon. **(A)** Body maximum intensity projection (MIP) image. **(B)** abdominal PET/CT.

Laboratory tests showed leukocytosis (20.79 × 10^9^/L), moderate anemia (69 g/L), increased percent of neutrophils (91.6%), increased C-reactive protein (CRP) (80.90 mg/L), procalcitonin (PCT) (5.87 ng/ml) and erythrocyte sedimentation rate (ESR) (106.0 mm/h) level, and mild hypoalbuminemia (31.0 g/L). Stool routine revealed Leukocyte ++++/HP, pus cell ++++/HP, stool blood was positive. Amoeba cysts were found after repeated stool examinations, but no trophozoites. Blood cultures, HBV, CMV-DNA, TORCH-IgM, G/GM, TB-IGRA, parasite antibody were negative. The plasma biochemistry of liver and kidney functions were normal. A total of three colonoscopes and biopsies were performed for the patient, and the results showed multiple irregular ulcers in the whole colon (Figure 1). Colonic biopsies diagnosed Epstein-barr virus-associated lymphoproliferative disorder, but the sample was inadequate for definitive diagnosis. Bone marrow examination showed the proliferation of hematopoietic cells were active, mainly granulocytes, and immature granulocytes increased. Findings of CT images of chest and neck were normal. Contrast-enhanced CT scan of the whole abdomen revealed multi-segmental intestinal wall thickening and enhancement (Figure 2A). 18F-FDG PET/CT demonstrated increased FDG uptake in the whole colon, bone marrow and spleen (Figure 3).

**Figure 4:**
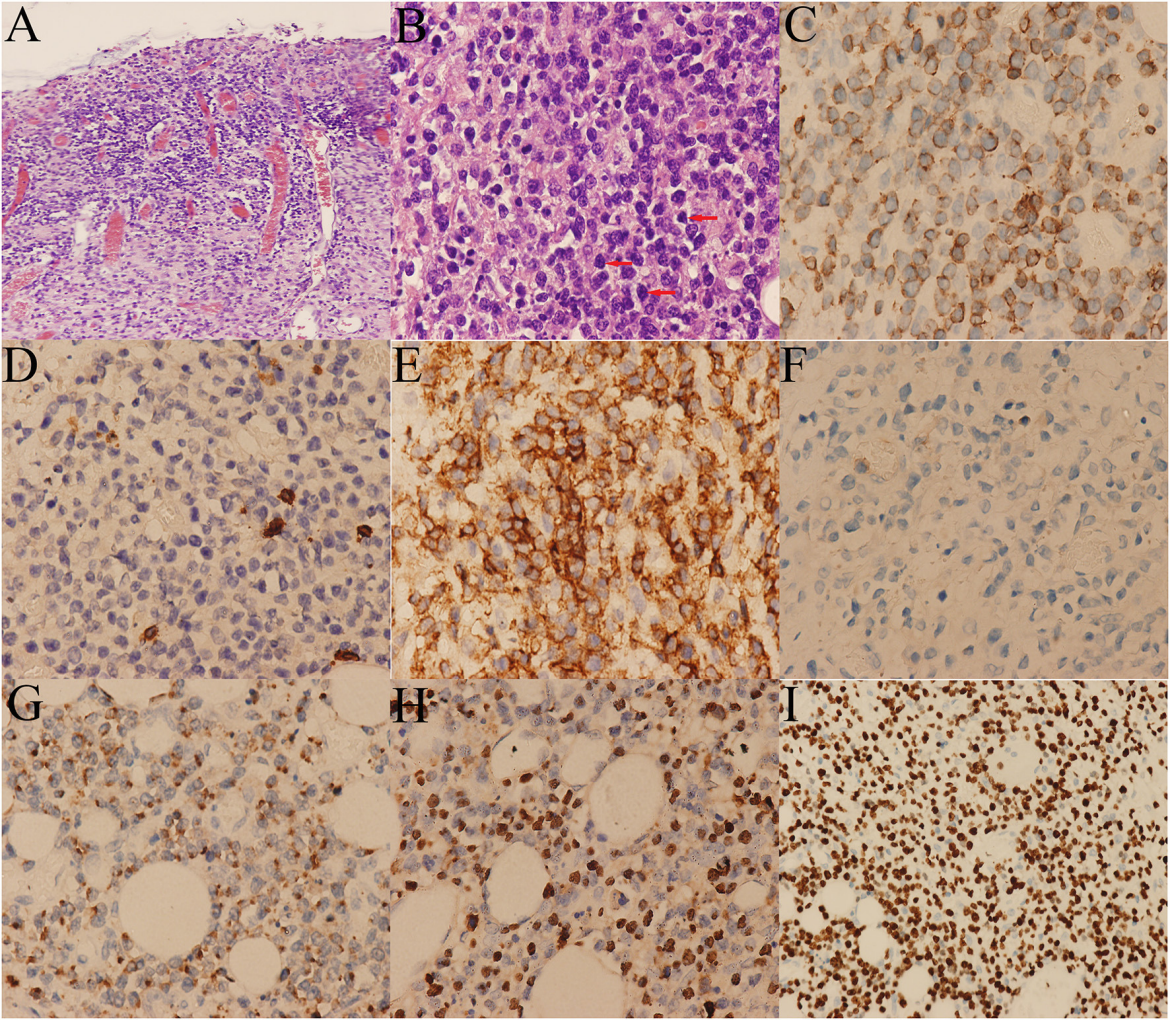
Histological and immunohistochemical pictures of the lymphoma. **(A)** The tumor cells infiltrated the whole wall of the colon. **(B)** Diffusely infiltrating of medium-sized tumor cells with irregular nuclei (arrows). Immunohistochemical stains showing the tumor cells are positive for **(C)** CD3, **(E)** CD43, **(G)** GB, and **(H)** EBER, and negative for **(D)** CD5, **(F)** CD56. **(I)** The Ki-67 labeling index was around 60%.

Treatment of the patient was provided with anti-infective (moxifloxacin, rifaximin, metronidazole and imipenem, vancomycin, and cefperazone-sulbactam, tigecycline were given successively). However, he still had diarrhea and recurrent fever, the body temperature fluctuated at 39–40°C. Blood cultures were still negative. No positive bacilli was found by acid fast staining and no DNA fragment of Mycobacterium tuberculosis was found by qPCR. During the hospitalization, the patient developed a fierce abdominal pain, CT indicated gastrointestinal perforation (Figure 2B). An emergency surgery was performed. During surgery, it was found that there were two 0.5 cm breaks in the ileocecal junction, a 3 cm break in the descending colon, three perforation holes of varying sizes were seen in the sigmoid colon with a diameter of about 0.5–1 cm, and three 0.5–4 cm breaks in the upper rectal segment 3 cm from the peritoneal reflection. The intestinal wall around the breaks was edematous and congestive. He underwent total colectomy and enterostomy. ENKTL was diagnosed for pathological diagnosis of surgical samples. Histologically, the tumor cells were medium in size with irregular nuclei (Figure 4B). There were mixed inflammatory infiltrated mainly including lymphocytes and plasmacytes. The tumor cells infiltrated the whole wall of the intestinal wall with ulcer, necrosis (Figure 4A). Immunohistochemical staining showed that CD3, CD2, CD43, granzyme B (GB), TIA-1 were positive, while CD20, CD5, CD7, CD4, CD8, CD56 were negative (Figures 4B-G). CD30 was focally positive. The Ki-67 labeling index was 60% (Figure 4I). EBV-encoded small RNA (EBER) analysis showed positive (Figure 4H). Gene rearrangement test found the low amplification peak of TR gene.

After recovering from surgery, the patient was treated with two courses of etoposide 50 mg and dexamethasone 5 mg (ED) regimen. Gemcitabine was added on the third day of the second course of therapy. Two weeks later, PD-1 monoclonal antibody 100 mg was used to replace ED regimen because his general condition and the expected chemotherapy tolerance were poor (A total of 3 times, each interval of 3 weeks). Fortunately, the patient was discharged the next day after the last treatment with PD-1 monoclonal antibody. By the time he was discharged from the hospital, his general condition improved significantly. PD-1 monoclonal antibody 100 mg was used on day 23, 56, 85 after discharged. To date, the patient has treated with a total of six courses of PD-1 monoclonal antibody. Eleven months follow-up was uneventful.

## Discussion

Primary intestinal NK/T cell lymphoma (PIENKTL) is an extremely rare and invasive tumor. Most of the patients had a poor prognosis and the average survival period was only 2.83–9.5 months. The most common site of involvement of PIENKTL is the colon, followed by the jejunoileum and ileocecum. The prevalence is higher in the third decade of life (5–8). A small number of patients with intestinal NK/T cell lymphoma will have a complication of perforation, including the case we present. Perforation mechanism of the intesitinal wall is not well-known, we hypothesized that might be related to the infiltration of mesenteric vascular and serous. Currently, few studies with small sample sizes have been reported so little is known about its clinical characteristics. The most common clinical symptoms reported in the studies (9, 10) include abdominal pain, diarrhea, fever, and intestinal bleeding. Clinical manifestations of our case were consistent with reported in studies. Subsequently, we reviewed and summarized the clinical characteristics, pathological features, and treatment approach of reported cases of PIENKTL (Table 1).

**Table 1:** Summary of the published cases of primary intestinal NK/T cell lymphoma.

**References**	**Age**	**Sex**	**Clinical manifestations**	**Location**	**Morphologic features**	**Immunophenotype**	**ISH of EBER**	**Treatment**	**OS (median OS)**
Duan et al. (11)	12	M	Abdominal pain, fever	Colon	Medium-sized atypical lymphoid cells with large areas of necrosis	CD2+, CD3+, CD4+, CD5+, CD8+, CD43+,CD56+, TIA-1+, GB+, Ki-67: 60%	Positive	Surgery	Within 1 month
Li et al. (12)	40	F	Loose stools, fever	Colon	Focal heterotypic lymphocyte infiltration?heterotypic lymphocyte clusters	CD2+, CD3+, CD5+, CD20+, CD34+, CD61+, CD79α TIA-1+, Ki-67: 40%	Positive	Methylprednisolone combined with Podexan and a salford enema	a few months
Lookzadeh et al. (13)	56	M	Fever, productive cough, dyspnea, Vomiting, chill	Terminal ileum	Infiltration of pleomorphic neoplastic lymphoid cells	CD3+, CD8+, CD30+, CD56+, LCA+, Ki-67: 70%	N/A	Surgery	N/A
Yang et al. (9)	15–72	M: 9; F: 4	Abdominal pain, gastrointestinal bleeding, diarrhea, fever	Small intestine: 4; large intestine: 9	The tumor cells were predominantly medium to small in size	CD3ε+, CD5+, CD56+, CD43+, TIA-1+, GB+, The Ki-67 index ranged from 30 to 70%	Positive	Surgery, chemotherapy, Surgery + chemotherapy	6 months
Wang et al. (10)	24–54	M: 6; F: 6	Abdominal pain, haematochezia, diarrhea, weight loss, fever	ileal: 1; ileocecum: 7; colon: 9; rectum: 5	Chronic mucosal/submucosal inflammatory infiltrates with clusters of medium- or small-sized lymphoid cells	CD3ε+, CD56+, TIA-1+, GB+, The Ki-67 index ranged from 5 to 80%	Positive	Surgery, chemotherapy, Surgery + chemotherapy	6.3 months
Tang et al. (6)	15–85	M: 14; F: 3	Abdominal pain, haematochezia, diarrhea	Small intestine: 5; ileocecum: 5; colon: 10	The tumor cells were small (23.5%), medium to small (41.2%) and medium (23.5%), medium to large (11.8%) in size	CD8+, CD30+, CD56+, TIA-1+, GB+	Positive	Surgery, chemotherapy, Surgery + chemotherapy	4.3 months
Yu et al. (14)	14–75	M: 37; F: 18	Abdominal pain, lower GI bleeding or fecal occult blood, fever	Small intestine: 28; ileocecum: 11; colon: 13; different intestinal segments: 3	The atypical lymphoid cells infiltrated, the tumor cells were displayed a mixed population of small, medium to large (43.6%), small to medium (32.7%), medium (21.8%), small (1.8%) in size	CD2+, CD4+, CD5+, CD7+, CD8+, CD30+, CD56+, TIA-1+, GB+, The Ki-67 index ranged from 50 to 90%	Positive	Surgery, chemotherapy, Surgery + chemotherapy	12.7 months
Chen et al. (15)	29	M	Abdominal pain, diarrhea, haematochezia, fever	Small intestine	The tumor cells were large in size	CD56+, Ki-67: 10%	Positive	Surgery + chemotherapy	20 d
Fang et al. (7)	24–68	M: 7; F: 3	Abdominal pain, diarrhea, haematochezia, fever	Small intestine	The tumor cells were small (20%), medium (20%), large (50%) in size, or mixed medium and large cells (10%)	CD2+, CD3+, CD30+, CD56+, TIA-1+, GB+, The Ki-67 index ranged from 40 to 90%	Positive	Surgery, chemotherapy, Surgery + chemotherapy	9.5 months
Aniwan et al. (16)	29	M	Abdominal pain, bloody diarrhea, fever, weight loss	Colon	Small lymphoid cells	CD3+, CD56+	Positive	Surgery	N/A
Zheng et al. (17)	37	M	Abdominal pain, bloody diarrhea, fever	Colon	Diffuse proliferation of small and medium sized atypical lymphoid cells	CD3ε+, CD56+, GB+	Positive	Surgery + chemotherapy	3 months
Mahuad et al. (18)	52	M	Fever	Colon	The tumor cells were medium to large in size	CD2+, CD56+, CD20+, GB+, Ki-67: 40%	Positive	Surgery + chemotherapy	2 months
Zheng et al. (5)	15–65	M: 16; F: 9	Abdominal pain, fever, weight loss, diarrhea, hematochezia	Small intestine: 8; ileocecum:8, large intestine: 16	The tumor cells were predominantly medium or large in size	CD3+, CD56+, GB+, The Ki-67 index ranged from 50 to 80%	Positive	Surgery, chemotherapy, surgery + chemotherapy	7 months
Panarelli et al. (19)	37	F	GI bleeding	Small intestine	The tumor cells were medium to large in size	CD2+, CD3+, CD7+, CD56+	Positive	Surgery	N/A
Wakabayashi et al. (20)	47	M	Abdominal pain, bloody stool, fever	Small intestine	Extensive diffuse infiltration by atypical lymphocytes in the lamina propria prosae	CD3+, GB+	Positive	Surgery + BMT	14 months
Kakimoto et al. (21)	73	M	N/A	Rectum	The tumor cells were large in size	CD3+, CD56+, GB+	Positive	Surgery	N/A
Moubayed et al. (22)	72/59	M	Abdominal pain, weight loss, diarrhea	Terminal ileum	The tumor cells were large in size	CD2+, CD3+, CD7+, CD8+, CD56+, TIA-1+	Negative	Surgery/surgery + chemotherapy	N/A

*F, female; M, male; ISH, in situ hybridization; EBER, EBV-encoded small RNA; OS, overall survival; TIA-1, T-cell restricted intracellular antigen 1; GB, granzyme B; N/A, not available; GI, gastrointestinal; BMT, bone marrow transplant*.

The study of Jiang et al. (8) found that if the patients were operated on before perforation, there was an improved prognosis, whereas patients who underwent surgery after perforation had a similar prognosis to patients who underwent no surgery. This implies the importance of early diagnosis and operation before perforation of PIENKTL. In clinical practice, the presence of PIENKTL should be alerted if the following situations occur: moderate-to-high grade fever occurs recurrently, CT showes thickening of multi-segment intestinal wall and enhancement of enlarged lymph nodes of mesentery, colonoscopy showes multiple, irregular, and variable-sized deep ulcers (23, 24).

However, early diagnosis of PIENKTL is frequently diffcult. Most patients are definitely diagnosed only after surgical resection of following complications. PIENKTL is easily misdiagnosed as inflammatory bowel disease (IBD) clinically. We herein summarize several possible reasons for the misdiagnosis. The clinical manifestations, endoscopic features, and imaging findings of IBD are similar to PIENKTL and compared with PIENKTL, IBD is a more common disease. Histologically, features of IBD, including crypt abscesses, anomalies in structure or number of glands, gangliocyte hyperplasia and even non-caseating granulomas, can be found in some cases of PIENKTL (10). In addition, it is difficult to distinguish the atypical lymphoid cells from normal reactive lymphocytes, a large amount of inflammatory infiltrates may obscure the relatively small number of tumor cells. Atypical lymphoid cells are usually clustered deep in the submucosa or break through the serous membrane, but mucosa involvement is uncommon (5). Therefore, we can't make a diagnosis easily if intestinal malignant lymphoma is suspected with negative histological evidence. Repeat endoscopy combined with deep biopsies may increase the possibility of definitive pathological diagnosis. If necessary, an exploratory laparotomy can be considered in time for early diagnosis.

Pathologically, PIENKTL should be differentiated from the other intestinal T cell lymphomas, including enteropathy associated T-cell lymphoma (EATL), monomorphic epitheliotropic intestinal T-cell lymphoma (MEITL), and peripheral T-cell lymphoma, not otherwise specified (PTCL, NOS). EATL formerly thought to be composed of two subtypes known as type I and type II. Type I EATL is now simply classified as EATL. It is strongly associated with coeliac disease. Type II EATL has been renamed MEITL. It shows no definite association with coeliac disease (25). Histologically, necrosis, and ulcer can be observed in PIENKTL and EATL, while necrosis is rare in MEITL. The tumor cells can infiltrate full-thickness of the intestinal wall both in PIENKTL, EATL, and MEITL. Epithelial involvement is common in EATL and MEITL, but not in PIENKTL. The tumor cells of PIENKTL are usually small to medium in size. The neoplastic cells of EATL are medium to large in size with prominent nucleoli, and they can have an immunoblastic or anaplastic appearance (6, 25–27). Most T cell and NK/T cell lymphomas have a common histological feature: irregular tumor cells of different shape or size mixed with inflammatory component. However, MEITL is an exception, for it shows with monotonous small tumor cells and few reactive cells. In addition, angiocentrity can usually be observed in PIENKTL and EATL, while it is absent in MEITL (6). Most MEITLs are positive for megakaryocyteassociated tyrosine kinase, which is diagnostically useful when expressed by >80% of cells (25). *In situ* hybridization for EBER plays a key role in differential diagnosis, which is negative in EATL, MEITL, and PTCL, NOS cases while positive in PIENKTL cases (6). The diagnosis of PTCL, NOS is based on the exclusion of all the other T cell lymphomas. Furthermore, secondary involvement of the intestine by nodal T cell lymphoma requires exclusion (6, 25). A definitive diagnosis of PIENKTL depends on immunohistochemistry. Compared to other sites of involvement, there is no difference in the immunophenotype of intestinal NK/T cell lymphoma. The tumor tissues express CD3ε, CD43, CD56, and cytotoxic molecules (such as TIA-1 and GB). Nuclear labeling for *in situ* hybridization for EBER is positive and T-cell receptor gene rearrangement (9). CD56 is an NK cell marker, and the studies (5, 14) showed that the positive frequency of CD56 in PIENKTL was 84–89.1%. Although it can help diagnose the PIENKTL, it is not specific for NK/T cell lymphoma. Few patients were negative for CD56 and the case we report was rare with lacking of CD56 expression.

Appropriate treatment of PIENKTL is very important to improve the prognosis. However, a standard treatment strategy for PIENKTL has not yet been established. Kim's study (28) found that patients who underwent surgery plus chemotherapy showed a relatively better survival prognosis than those treated with chemotherapy alone. To date, there is no standard chemotherapeutic regimen for PIENKTL. In the previous studies (8, 14), the chemotherapy regimens of PIENKTL were varied. Chemotherapy with a DICE (dexamethasone, ifosfamide, cisplatin, and etoposide), CHOP (cyclophosphamide, doxorubicin, vincristine and prednisone) or L-asparaginase/peg-asparginase-containing regimen was most often employed. So far, not much information is available on the role of radiotherapy in PIENKTL.

## Conclusions

Early diagnosis of PIENKTL is extremely difficult, most patients are diagnosed at an advanced stage, resulting in a poor prognosis. It is difficult to obtain a definitive diagnosis by limited histopathological data offered by endoscopic biopsy, so repeat endoscopy combined with deep biopsy is necessary. When multiple biopsies still can't make a definite diagnosis but intestinal malignant lymphoma is highly suspected, exploratory laparotomy should be performed, so as not to delay the diagnosis.

## Data Availability Statement

The original contributions presented in the study are included in the article/supplementary material, further inquiries can be directed to the corresponding author.

## Ethics Statement

Written informed consent was obtained from the individual(s) for the publication of any potentially identifiable images or data included in this article.

## Author Contributions

BW designed the report. YZ wrote the paper. WL performed the histopathological examination of the tumors and provided the pictures of tumor pathology. XZ provided and analyzed the pictures of PET/CT. All authors read and approved the final manuscript.

## Conflict of Interest

The authors declare that the research was conducted in the absence of any commercial or financial relationships that could be construed as a potential conflict of interest.
